# Meaningful Activity for Residents with Dementia in Assisted Living: Results From the MAC-4-BSD Pilot Study

**DOI:** 10.1093/geroni/igaf122.899

**Published:** 2025-12-31

**Authors:** Sarah Holmes, Elizabeth Galik, Barbara Resnick, Sorah Levy, Erin O’Brien, Stephanie McCorvey, Merve Gurlu, Shijun Zhu

**Affiliations:** University of Maryland School of Nursing, Baltimore, Maryland, United States; University of Maryland School of Nursing, Baltimore, Maryland, United States; University of Maryland, Baltimore, Maryland, United States; University of Maryland, Baltimore, Maryland, United States; University of Maryland School of Nursing, Baltimore, Maryland, United States; University of Maryland at Baltimore, Baltimore, Maryland, United States; University of Maryland School of Nursing, Baltimore, Maryland, United States; University of Maryland School of Nursing, Baltimore, Maryland, United States

## Abstract

Engagement in meaningful activity is associated with increased sense of purpose, improved mood, and decreased behavioral and psychological symptoms. Despite these benefits, there are limited opportunities for assisted living (AL) residents with dementia to engage in activities that are personally valued and meaningful. This study evaluated the feasibility and preliminary efficacy of a theory-based approach, Meaningful Activity for Managing Behavioral Symptoms of Distress (MAC-4-BSD). Using a cluster randomized controlled trial with repeated measures design, 66 residents with dementia from 5 AL communities were included. AL communities were randomized to either MAC-4-BSD or education-only control group. Outcomes were evaluated at baseline and 4 months. It was hypothesized that exposure to MAC-4-BSD will demonstrate increased meaningful activity engagement, improved quality of life, and reduced behavioral symptoms compared to the control group. Descriptive statistics and linear mixed models were used. Most participants were female (n = 49, 74%), and White (n = 58, 88%) with a mean age of 85 years (SD = 8.4). MAC-4-BSD was feasible to implement based on evidence of delivery and receipt of the intervention. From baseline to 4 months, residents exposed to MAC-4-BSD had significant improvements in meaningful activity engagement (b = 7.8, p=.008, Cohen’s d=.74) and decreased number of behavioral symptoms (b=-2.3, p=.010, Cohen’s d=-.79) relative to the control group. These findings demonstrate that engagement in meaningful activity is a promising approach that is feasible and can help to address behavioral and psychological symptoms for AL residents with dementia. Future research is needed to support the long-term sustainability and adoption of MAC-4-BSD in AL communities.

